# Digital *E. coli* Counter: A Microfluidics and Computer Vision-Based DNAzyme Method for the Isolation and Specific Detection of *E. coli* from Water Samples

**DOI:** 10.3390/bios12010034

**Published:** 2022-01-10

**Authors:** Sakandar Rauf, Nouran Tashkandi, José Ilton de Oliveira Filho, Claudia Iluhí Oviedo-Osornio, Muhammad S. Danish, Pei-Ying Hong, Khaled N. Salama

**Affiliations:** 1 Sensors Laboratory, Advanced Membranes & Porous Materials Centre (AMPMC), Computer, Electrical, and Mathematical Sciences and Engineering (CEMSE) Division, King Abdullah University of Science and Technology (KAUST), Thuwal 23955-6900, Saudi Arabia; sakandar.rauf@kaust.edu.sa (S.R.); nouran.tashkandi@outlook.com (N.T.); jose.deoliveirafilho@kaust.edu.sa (J.I.d.O.F.); 2 Water Desalination and Reuse Center, Division of Biological and Environmental Science and Engineering, King Abdullah University of Science and Technology (KAUST), Thuwal 239455-6900, Saudi Arabia; claudia.oviedoosornio@kaust.edu.sa (C.I.O.-O.); peiying.hong@kaust.edu.sa (P.-Y.H.); 3 Department of Computer Science, Information Technology University (ITU), Arfa Software Technology Park, Lahore 40050, Pakistan; msds20004@itu.edu.pk

**Keywords:** DNAzyme, *E. coli*, microfluidics, water quality, fluorescence detection, computer vision

## Abstract

Biological water contamination detection-based assays are essential to test water quality; however, these assays are prone to false-positive results and inaccuracies, are time-consuming, and use complicated procedures to test large water samples. Herein, we show a simple detection and counting method for *E. coli* in the water samples involving a combination of DNAzyme sensor, microfluidics, and computer vision strategies. We first isolated *E. coli* into individual droplets containing a DNAzyme mixture using droplet microfluidics. Upon bacterial cell lysis by heating, the DNAzyme mixture reacted with a particular substrate present in the crude intracellular material (CIM) of *E. coli*. This event triggers the dissociation of the fluorophore-quencher pair present in the DNAzyme mixture leading to a fluorescence signal, indicating the presence of *E. coli* in the droplets. We developed an algorithm using computer vision to analyze the fluorescent droplets containing *E. coli* in the presence of non-fluorescent droplets. The algorithm can detect and count fluorescent droplets representing the number of *E. coli* present in the sample. Finally, we show that the developed method is highly specific to detect and count *E. coli* in the presence of other bacteria present in the water sample.

## 1. Introduction

Water contamination is a global health issue. According to WHO, approximately 2 billion people worldwide use drinking water contaminated with feces, a major transmission source of different diseases such as diarrhea, cholera, typhoid, and polio [1]. Water contaminants are classified into biological, inorganic, organic, and radiological contaminants [2]. While there are commercially available sensors for detecting inorganics (e.g., calcium and chloride ions) [3] and organics (e.g., total organic carbon) [4], biosensors for real-time or continuous monitoring of biological contaminants are still in an earlier stage of development [5,6]. The current ways to detect biological contaminants in the water are time-consuming and do not facilitate data-driven decision-making to protect public health [5,6]. For example, coliforms such as *E. coli* can be used as an indicator of microbiological contamination to assess the water quality [7]. The conventional method of *E. coli* detection incurs a long culture time, and observation of phenotypic traits on enzymatic agar media can be prone to false-positive or negative results [8,9]. Therefore, various new methods have been developed for the rapid detection of *E. coli,* such as nucleic acid-based methods (e.g., DNA microarray and polymerase chain reaction (PCR)) and immune-based methods (e.g., enzyme-linked immunosorbent assays (ELISA) and lateral flow immunoassay) [10]. ELISA-based methods have been used to detect a wide variety of pathogens; conversely, they are prone to a low sensitivity and antigen-cross reactivity, decreasing the assays’ overall efficiency [10]. PCR-based methods can achieve quantitative detection in the presence of a lower concentration of bacteria, although these methods require bacterial DNA extraction and involve lengthy and complicated procedures [9,11].

DNAzyme biosensors have emerged as a new strategy to detect *E. coli* that has the potential of overcoming the limitations associated with the currently available *E. coli* detection methods. DNAzyme reagents consist of short single-stranded DNA sequences that can fold into complex tertiary structures in the presence of a particular target or cofactor, resulting in a catalytic activity that can be used as a basis for signaling output to denote the presence of the intended target or cofactor [12]. Due to the ease in modification of DNAzyme, several fluorescence-quencher-based strategies have been developed [13]. In particular, for detecting *E. coli*, different DNAzyme-based methods have been reported in recent years. A graphene-DNAzyme hybrid material-based fluorescence biosensor was developed to detect *E. coli* [14]. In another study, agarose beads were modified with DNAzyme and loaded into a syringe-based biosensor [15]. Upon reaction with the *E. coli* containing mixture, the fluorescent cleavage fragment was released from the beads and was filtered onto a nitrocellulose paper for fluorescence detection [15].

Droplet generation using microfluidics is a well-known, established technique and has many applications in chemical and biological sciences [16]. Several new and improved methods have been reported to generate droplets and have demonstrated applications in developing different biological assays [17,18,19]. A microfluidics-based strategy was combined with a DNAzyme sensor to detect *E. coli (E. coli* K12) from unprocessed blood samples [20]. The DNAzyme and *E. coli* were confined inside the droplets, which act as a microcarrier. Due to the droplets’ small volume (picoliters), the concentration of the released target from the bacterial cell lysis becomes significantly higher than the bulk solution-based method, which helps generate a readable fluorescent signal even with a low activity rate of the DNAzyme inside the droplets [20]. However, this strategy used a custom-made expensive confocal microscopy system and was limited to small volume samples (2 mL). Generally, acceptable drinking water quality requires zero *E. coli* or Coliform in 100 mL water [21]. Therefore, more efforts are needed to develop droplet-based DNAzyme sensor platforms or other biosensor platforms that can process large sample volumes (100 mL) to detect *E. coli* in the water samples.

Herein, we report a highly specific microfluidics-based DNAzyme sensor method that can detect and count the number of *E. coli* in a water sample. The *E. coli* from the water sample were first isolated in single water droplets that contain a DNAzyme sensor, which, upon heating, give a fluorescence signal, indicating the presence of *E. coli* in the water droplet. The droplets were produced using the traditional flow-focusing microfluidic approach [22] to encapsulate *E. coli* from the water sample. We developed a fluorescent droplet digital counter using a simple computer vision method that counts the fluorescent droplets passing through a microfluidic channel. This is the first report showing the potential for detecting *E. coli* in water samples, combining a DNAzyme sensor, droplet microfluidics, and computer vision methods. The proof of concept described in these studies shows the potential of real-time monitoring of *E. coli* and other microbial contaminants present in large water samples. It would facilitate the development of a smart and automated water quality assessment system in the future. 

## 2. Materials and Methods

### 2.1. Bacterial Strains

*E. coli* DSM 1103 (German Collection of Microorganisms and Cell Cultures DMSZ, Braunschweig, Germany) and other non-*E. coli* bacterial strains (*Serratia* (EPA 74), *Klebsiella* (EPA 193), and *Morganella* (MA 35)) were isolated from samples described in previous studies [23,24]. The bacteria was first streaked on LB agar and incubated for 24 h at 37 °C. A single colony of roughly 1 mm in diameter was picked from the LB plate and used for the inoculation of the bacteria. The single colony was inoculated in 10 mL SOB broth (Hanahan’s broth) and was incubated for 12 h in a shaker incubator (37 °C). In the case of all the bacterial cultures, the optical density (OD) at 600 nm (OD600) was measured. For the analysis of the samples, 10 µL from the culture broth (OD600 = 1) containing *E. coli* was diluted to 1 mL of deionized water (final concentration of *E. coli* ≅ 8 × 10^7^/mL) [25]. In the case of specificity studies, 10 µL from each culture broth of EPA 74, EPA 193, and MA 35, with or without *E. coli* (OD600 adjusted to 1), was diluted to 1 mL using deionized water (final concentration of each bacteria ≅8 × 10^7^/mL). It is important to note that the number of bacteria (*E. coli* and other bacteria) values are not absolute and are based on the calculation from the absorbance values [25]. 

### 2.2. Microfluidics-Based Fluorescence Measurement Set-Up

**Figure 1:**
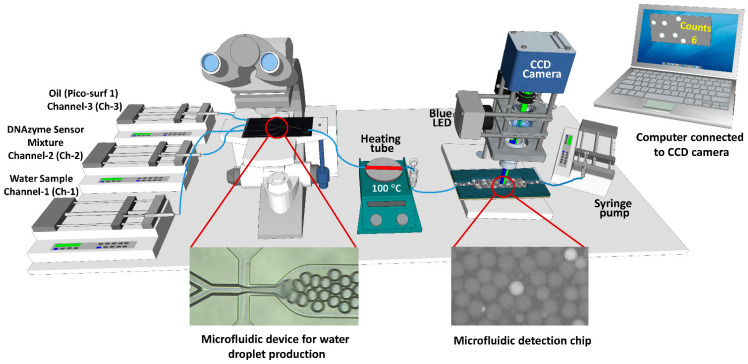
Scheme of the microfluidic set-up for the isolation and detection of *E. coli*.

**Figure A1:**
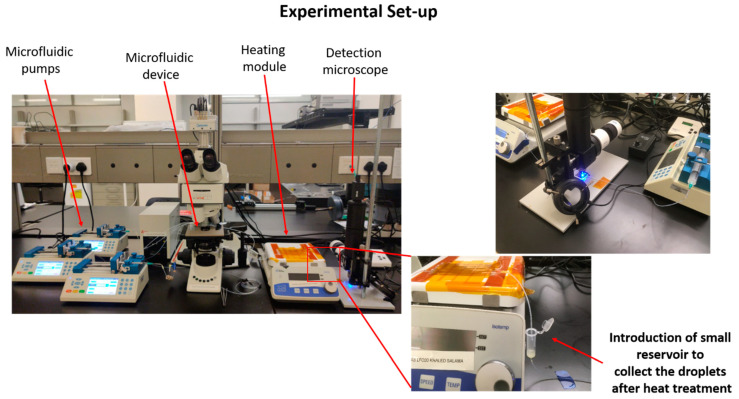
Photograph of the scheme of the microfluidic set-up for the isolation and detection of *E. coli*.

The microfluidic set-up for producing and detecting the fluorescence droplets is shown in Figure 1 and Figure A1 (in Appendix A). The set-up contains two microfluidic devices. The first is a two-reagent droplet chip with a 50 µm etch depth and a hydrophobic coating (Dolomite Microfluidics, UK) to produce water droplets. The flow rate of the water sample (Channel-1 (Ch-1)) and DNAzyme mixture (Channel-2 (Ch-2)) was maintained at 2.5 µL/min for each channel. Different flow rates (5, 7.5, and 10 µL/min) of the oil phase (Channel-3 (Ch-3)) were used to produce water droplets and to determine the optimized flow rate for the oil phase that allows for good segregation and encapsulation of a single bacterial cell within each droplet. The oil phase consists of Pico-Surf™ (5% (*w*/*w*) in Novec™ 7500). The outlet of the chip was connected to a stainless steel tube (Figure 1, shown in red) with a length of 11 cm made from a 21 gauge needle, and was fixed on a hotplate for heat treatment. The stainless steel tube was covered with polyimide tape for insulation and to help maintain the temperature inside the tube. The droplets were then collected into an Eppendorf tube with a hole at the bottom, which acted as an inlet to withdraw the droplets from the Eppendorf tube using another microfluidic pump. The droplets passed through the second microfluidic device for detection under the custom-made fluorescence microscope. The dimensions of the second microfluidic device channel had a width of 1 mm, height of 50 µm, and length of 20 mm. The second microfluidic device was fabricated as follows. A single microfluidic channel was designed using Coral Draw software. The design was fabricated on a polymethyl methacrylate (PMMA) sheet (thickness-75 µm) using a Universal laser VLS 3.50 laser system to make the template for polydimethylsiloxane (PDMS) structures. The design was bonded on another flat PMMA sheet using heat and pressure (120 degrees, 50 Lbs). The PDMS mixture was prepared (1:10 ratio), poured onto the PMMA template, and cured at 65 °C. After 2.5 h, the solid PDMS was carefully cut and removed from the template and was bonded on a glass slide after being treated with oxygen plasma for 25 s. The device was placed on a hot plate (80 °C) for 15 min to strengthen the binding between the PDMS and glass. The first microscope is a commercially available Leica DM 3000 microscope (Leica Microsystems, Wetzlar, Germany) equipped with a high-speed color camera (CP70-16-M-148, Optronis GmbH, Kehl, Germany) to observe and monitor the production of droplets. The second microscope is a custom-made fluorescence microscope to collect the fluorescence signal from the DNAzyme sensor. This microscope contains a blue LED source (PE100, 470 nm), 10× objective lens, and a GFP filter set for the excitation and collection of fluorescence signals emitted from the DNAzyme sensor. The videos were collected using a CCD camera (Grasshopper3 USB3, FLIR, Wilsonville, OR, USA) connected to the fluorescence microscope. The average diameter of the droplets was calculated by processing the optical images of the droplets using Image J software. The data obtained from the Image J software were plotted as a histogram using Origin software.

### 2.3. Preparation of DNAzyme Sensor

A trans-acting DNAzyme probe [23,26] was used to detect *E. coli* in the water samples in this study. The DNAzyme probe consists of two oligonucleotides, FS1 and EC1T. The oligonucleotide FS1(5′-ACTCTTCCTAGC/iFluorT/rA/iDabdT/GGT TCG ATC AAG A-3′) is functionalized with a fluorophore (Emission 538 nm) and a quencher. The EC1T oligonucleotide (5′-GAT GTG CGTTGT CGA GAC CTG CGA CCG GAA CAC TAC ACT GTG TGGGGA TGG ATT TCT TTA CAG TTG TGT G-3′) binds with a particular target present in the crude intracellular material (CIM) of *E. coli* and cleaves the FS1 due to the catalytic activity of the EC1T. When FS1 is cleaved due to the catalytic activity, the fluorophore and quencher dissociate, leading to a fluorescent signal [23,26]. Aguirre et al. [26] reported the optimized conditions for the catalytic activity of the DNAzyme to detect *E. coli* from CIM by studying different parameters such as temperature, pH, the effect of different divalent ions, and cleavage activity of EC1T. The optimized EC1T/FS1 ratio (50:1) reported by Aguirre et al. [26] was used in the current study. It is important to mention that the EC1T/FS1 ratio (50:1) suggests that the DNAzyme has a low rate activity, and, therefore, requires an excess of the cleaving unit (EC1T) vs. the cleaved unit (FS1). Based on the optimized conditions reported by Aguirre et al. [26], 500 µL of DNAzyme reaction mixture contains 50 mM HEPES pH 7.5, 150 mM NaCl, 15 mM BaCl_2_, 0.5 µM FS1, and 25 µM EC1T. Before use, the reaction mixture was centrifuged at 13,000 rpm for 8 min to remove any aggregates.

### 2.4. Counting of Fluorescent Droplets

The code to detect and count fluorescent droplets was written in Python software and was trained using the TensorFlow platform. The algorithm developed in this study takes each frame from the video and processes it using the SSD (Single Shot MultiBox Detector) framework model [27]. The trained model returns the detected droplets from each frame, and they are counted once their centroid reaches the region of interest (ROI), an set to one-half of the frame’s height. In all the frames, a bounding box encloses the detected droplets and informs the confidence in percent of the neural network. By using an IoU (intersection over union) equal to 0.5, the accuracy of the object identifier is 95.4%. The estimator of this work takes 22 milliseconds to identify the droplets and takes 24.4 min to be trained based on transfer learning of the SSD MobileNet V2 Feature Pyramid Network. Furthermore, the counting of the droplets can be tunned by setting the minimum acceptable confidence level. The trained model can be used in real-time detection from the camera stream or post-processing using recorded videos.

## 3. Results and Discussion

**Figure 2:**
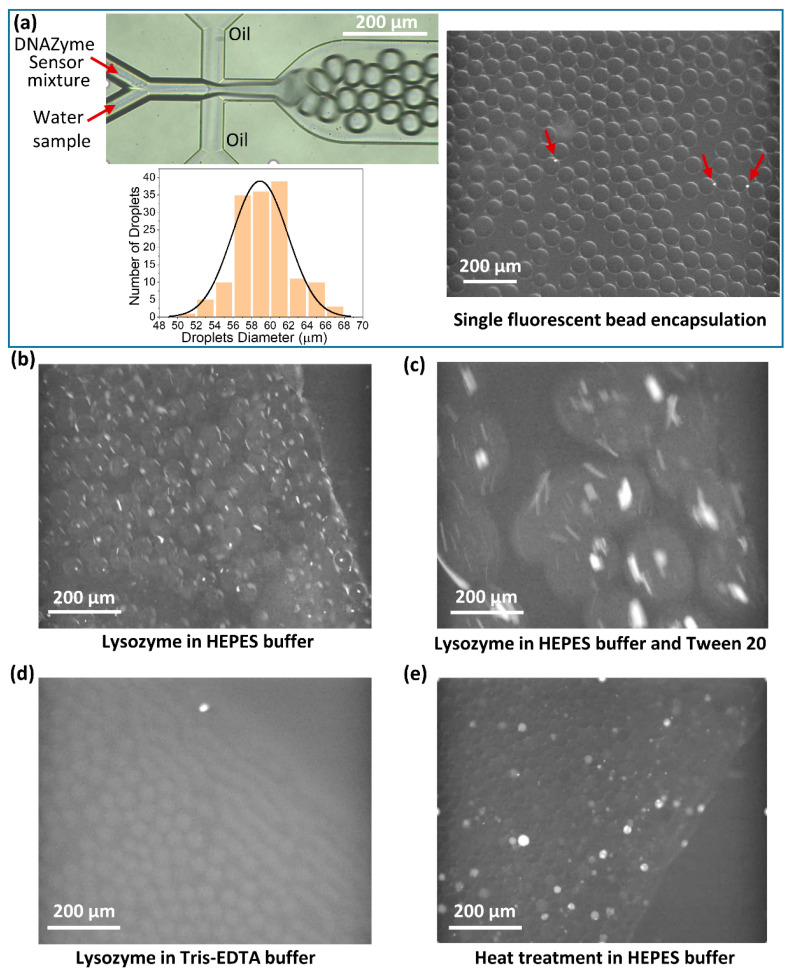
(**a**) Production of water droplets using the microfluidic device and encapsulation of fluorescent beads into the droplets. (**b**–**d**) Lysozyme (1 mg/mL) used in different buffer systems for bacterial cell lysis. (**e**) Cell lysis in HEPES buffer due to heat treatment at 105 °C.

Figure 1 and Figure A1 (in Appendix A) show the microfluidic set-up to generate water droplets that can encapsulate a single *E. coli* in each droplet and detect the presence of *E. coli*. First, the *E. coli* was isolated in the water droplets in this detection method. Then, after heating the emulsion at a higher temperature, DNAzyme produced a fluorescent signal indicating the presence of *E. coli*. Figure 2a (Video S1, Supplementary Materials) shows the production of water droplets. A commercially available droplet generator microfluidic device was used to make water droplets in oil. The average size of the droplets was found to be 59 ± 3 µm. To test whether a single *E. coli* can be encapsulated in each droplet, 1 µm streptavidin coated dragon green fluorescent polystyrene beads (Bangs laboratories, Inc., Fishers, IN, USA) were dispersed in deionized water and encapsulated in the water droplets. The size of the fluorescent beads was comparable to the size of the *E. coli* (typical size: 0.5 µm × 2.0 µm) [28]. It can be seen (Figure 2a and Video S2, Supplementary Materials) that single beads were encapsulated inside the water droplets, confirming that the size of the droplets was sufficient to encapsulate a single *E. coli*. We also found that bead concentration less than 10^4^/mL (data not shown) gave good segregation of one bead per droplet. Bead concentration above 10^4^/mL might lead to more than one bead per droplet. Therefore, the probability of encapsulation of more than a single *E. coli* cannot be ignored for higher concentrations of *E. coli*. There may be events where more than one *E. coli* may be encapsulated in one droplet.

Typically, lysozyme is used as a bacterial cell lysis agent in combination with different microfluidic devices [29]. Figure 2b–d shows the use of lysozyme in different buffer systems, along with the DNAzyme mixture. It can be seen that in the presence of lysozyme in the HEPES buffer, aggregates were formed in each droplet, which corresponds to the aggregation of DNAzyme probes. The fluorescent signal also indicates that lysozyme partially or completely cleaved the fluorescent probe (FS1) in the DNAzyme mixture resulting in the fluorescent signal (Figure 2b and Video S3, Supplementary Materials). Adding Tween 20 (0.05%) to the reaction mixture in the HEPES buffer produced even larger droplets and aggregates with the fluorescent signal (Figure 2c and Video S4, Supplementary Materials). When another buffer system, Tris-EDTA, was used, the reaction mixture appeared milky and turbid, and after centrifugation to remove large aggregates, no fluorescence signal was observed (Figure 2d and Video S5, Supplementary Materials). All of these experiments indicated that using lysozyme and the DNAzyme mixture used in this study was not suitable for bacterial cell lysis and the production of fluorescent signals in the presence of *E. coli*. Figure 2e (Video S6, Supplementary Materials) shows the heat treatment (105 °C) of the droplets containing *E. coli* after passing through the stainless steel tube (shown as red in Figure 1). It can be seen that the droplets that contained *E. coli* gave a bright fluorescent signal; however, the emulsion was not stable, and heat treatment produced small and large size droplets. In addition, bubbles were formed due to the emulsion heating at a higher temperature, making the emulsion flow move back and forth, as shown in Video S6 (Supplementary Materials). This effect was not suitable for reading the fluorescent signal from the droplets in a continuous manner. To overcome this problem, we introduced an Eppendorf reservoir to store the emulsion after heat treatment, which was simultaneously withdrawn towards the detection microscope using a syringe pump (Figure 1 and Figure A1, in Appendix A). This arrangement allows for the withdrawal of the emulsion at a constant flow rate (5 µL/min), which helps in the smooth readout of the fluorescent droplets containing *E. coli*. 

**Figure 3:**
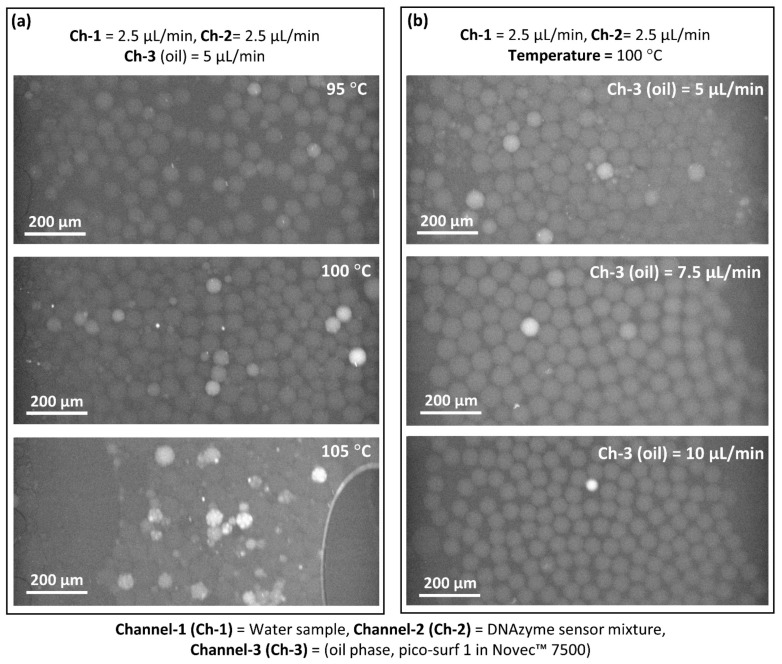
(**a**) Heat treatment of the water droplets at different temperatures to lyse the bacteria encapsulated inside the droplets. (**b**) Optimization of the flow rate of the oil phase while maintaining the aqueous medium flow rates constant. The approximate concentration of *E. coli* (number of *E. coli*/mL) used was ≅8 × 10^7^/mL.

As shown in Figure 1, the as-produced droplets directly pass through the heating stainless tube (shown red) placed on the hotplate. Therefore, the change in temperature and flow rate of the fluids are important parameters to optimize. Figure 3a shows the heat treatment of the droplets at different temperatures while keeping the flow rate of all the fluids constant. It can be seen that heating at 95 °C (Figure 3a and Video S7, Supplementary Materials), a weak fluorescent signal was observed in the droplets, which cannot be recognized distinctively as the droplets containing *E. coli* compared to the empty droplets (droplets without *E. coli*). This may be due to the insufficient bacterial cell lysis at this temperature. Typically, in the case of bulkbacterial lysis, heating at 65 °C for 30 min was enough for lysis [23]. However, higher temperatures are needed as the fluid passes through the heating tube in a continuous flow manner. Heating at 100 °C gave a clear detectable fluorescent signal compared with the empty droplets (Figure 3a and Video S8, Supplementary Materials). Heating at a higher temperature, 105 °C (Figure 3a and Video S9, Supplementary Materials), resulted in larger and smaller droplets due to the instability of the emulsion at this higher temperature. Therefore, 100 °C was selected as the optimum temperature.

After optimizing the lysis temperature, the flow rate is another critical parameter to be optimized. Figure 3b shows the effect of the change in the flow rate of the oil phase while keeping the water sample and DNAzyme flow rates constant. At a 5 µL/min flow rate of the oil phase (Figure 3b and Video S10, Supplementary Materials), although the fluorescent signal for the presence of *E. coli* can be identified easily compared to the empty droplets, tiny droplets can be seen, indicating that the emulsion was unstable at this flow rate after heat treatment. When the flow rate of the oil phase changed to 7.5 µL/min (Figure 3b and Video S11, Supplementary Materials), a uniform fluorescent signal was obtained for the *E. coli* containing droplets compared to the empty droplets. At a higher flow rate of 10 µL/min (Figure 3b and Video S12, Supplementary Materials), fewer fluorescent droplets can be seen, though the droplets’ become smaller in size than for the 7.5 µL/min flow rate. This indicates that at a 10 µL/min flow rate, the emulsion moved through the heating tube much faster than for the 7.5 µL/min flow rate, resulting in incomplete bacterial cell lysis. Therefore, the optimized parameters to detect *E. coli* using the DNAzyme mixture were heat treatment at 100 °C and an oil phase flow rate of 7.5 µL/min. 

**Figure 4:**
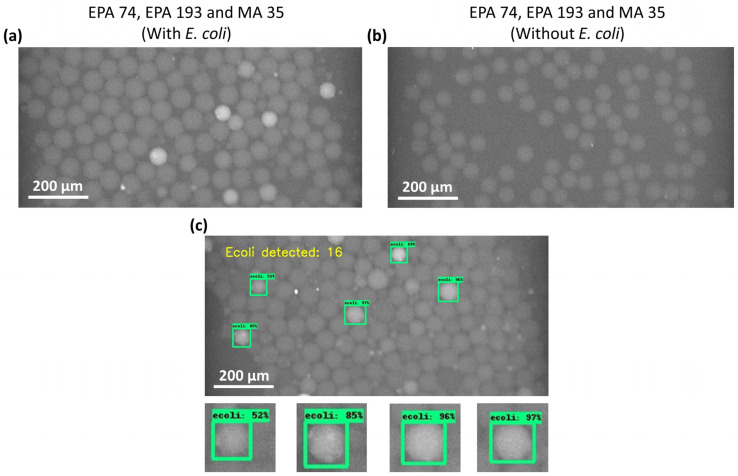
Specificity of the DNAzyme sensor. (**a**) Snapshot from the video obtained for the sample containing *E. coli* in the presence of other bacteria ((*Serratia* (EPA 74), *Klebsiella* (EPA 193), and *Morganella* (MA 35)). The approximate concentration (number of bacteria/mL) of each bacteria used was ≅8 × 10^7^/mL. (**b**) Snapshot from the video obtained for the sample without *E. coli* in the presence of other bacteria ((*Serratia* (EPA 74), *Klebsiella* (EPA 193), and *Morganella* (MA 35)). (**c**) Counting of the fluorescent droplets using the algorithm developed in Python. The algorithm tags the fluorescent droplets with a unique ID, and counts the fluorescent droplets.

DNAzyme sensors allow for highly specific detection of *E. coli* in the presence of other bacteria [20,23,26,30]. To verify specificity, we ran the samples containing three other bacteria ((*Serratia* (EPA 74), *Klebsiella* (EPA 193), and *Morganella* (MA 35)) with or without *E. coli* through our system. Figure 4a,b shows the fluorescent readout in the presence and absence of *E. coli* in the samples. It can be seen that *E. coli* can be detected as complete bright droplets in the presence of other bacteria in the sample (Figure 4a and Video S13, Supplementary Materials). However, we observed tiny bright aggregates inside some of the droplets, which may be due to the non-specific interaction between the DNAzyme and bacteria other than *E. coli* present in the droplets. The sample without *E. coli* did not give clear and bright fluorescent droplets indicating the absence of *E. coli* (Figure 4b and Video S14, Supplementary Materials). Only tiny bright aggregates can be seen inside the droplets confirming our previous observation in Figure 4a. This data show that the DNAzyme sensor under these conditions is highly specific and can detect *E. coli* in the presence of other bacteria.

The next step is the counting of *E. coli*. For this purpose, we combined a computer vision strategy to develop algorithms for detecting and counting fluorescent droplets from the videos taken during the experiment. Figure 4c and Video S15 (Supplementary Materials) show the counting of the droplets using the algorithm. The algorithm tags the fluorescent droplets in the presence of other non-fluorescent droplets and counts them. The tagged droplets that failed to keep more than 50% of the confidence level throughout the frames were not counted. The snapshot in Figure 4c shows that the algorithm tagged five fluorescent droplets and the number of *E. coli* counted at the time of the snapshot. The developed algorithm can detect and count the fluorescent droplets in the samples with and without *E. coli* (Videos S15 and S16, Supplementary Materials). Each fluorescent droplet indicates the presence of *E. coli* entrapped inside the droplet. Video S16 (Supplementary Materials) shows that in the sample without *E. coli* (zero *E. coli* added to the sample), the algorithm hardly detected any fluorescent droplets compared to the sample containing *E. coli*, indicating the absence of *E. coli*. These results demonstrate the high specificity of the current method. The addition of algorithm counting in this study can help minimize the errors incurred by manually counting fluorescent droplets.

## 4. Conclusions

We have developed a novel method based on microfluidics, DNAzyme, and computer vision to detect and count *E. coli* in water samples. The heating of water droplets at higher temperatures containing single *E. coli* lysed the individual bacterial cell encapsulated inside the water droplets, turning on the fluorescence signal by the DNAzyme sensor mixture. We demonstrated a straightforward solution of injection of the sample and counted the *E. coli* present in the water sample to test the water quality. Although we did not run a calibration curve with *E. coli*, we ran a calibration test with fluorescent beads of the same size as *E. coli*. We tested beads over a range of approximately 10^3^ to 10^5^ during those experiments and determined that 10^4^ is the best range for discrete segregation of bacteria-approximating particles per droplet. We also observed that the software image is able to identify the ca. 100 beads that are present within the sorted volume in its discrete form. As we did not further challenge the system to any range lower than 100, we tentatively state that the limit of detection (LOD) of this entire system is estimated at 100 bead particles per 50 µL of volume (the volume sorted through our system). Further improvements to the detection sensitivity can be made by improving the image recognition software and sorting rates, and ensuring sampling processing step that involves the concentration of large water volumes to the 50 µL needed to sort through the system can have a maximal recovery yield. 

The detection strategy reported here shows a proof of concept study to detect and count *E. coli* in water samples. However, further studies are needed to test the system for different concentrations of *E. coli* in varying types of water samples (e.g., tap water and recreational water), and verify the *E. coli* numbers by comparing them with a standard *E. coli* counting method. Combining the microfluidics and DNAzyme method with the computer vision strategy allowed for the detection and counting of *E. coli* from the videos. More efforts in this direction can help develop a real-time detection and counting algorithm that counts *E. coli* as they pass under the fluorescence detection microscope, potentially processing large water samples (100 mL water sample) for *E. coli* and/or other microbial contaminants’ detection. We envisage that the proof of concept study presented here will lead to developing smart water quality assessment systems in the future. 

## Data Availability

Data are contained within the article or in the supplementary material.

## Supplementary Materials

The following are available online at https://www.mdpi.com/article/10.3390/bios12010034/s1: Video S1: Droplet production. Video S2: Single bead encapsulation. Video S3: Lysozyme in HEPES at room temperature. Video S4: Lysozyme in HEPES buffer with Tween 20. Video S5: Lysozyme Tris EDTA buffer. Video S6: High-temperature treatment in HEPES. Video S7: Heat treatment at 95 °C. Video S8: Heat treatment at 100 °C. Video S9: Heat treatment at 105 °C. Video S10: 5 µL per min flow rate. Video S11: 7.5 µL per min flow rate. Video S12: 10 µL per min flow rate. Video S13: With *E. coli*. Video S14: Without *E. coli*. Video S15: *E. coli* counting. Video S16: Without *E. coli* counting.
